# Visual Feature Integration Indicated by pHase-Locked Frontal-Parietal EEG Signals

**DOI:** 10.1371/journal.pone.0032502

**Published:** 2012-03-09

**Authors:** Steven Phillips, Yuji Takeda, Archana Singh

**Affiliations:** 1 Mathematical Neuroinformatics Group, Human Technology Research Institute, National Institute of Advanced Industrial Science and Technology (AIST), Tsukuba, Ibaraki, Japan; 2 Cognition and Action Research Group, Human Technology Research Institute, National Institute of Advanced Industrial Science and Technology (AIST), Tsukuba, Ibaraki, Japan; University of British Columbia, Canada

## Abstract

The capacity to integrate multiple sources of information is a prerequisite for complex cognitive ability, such as finding a target uniquely identifiable by the conjunction of two or more features. Recent studies identified greater frontal-parietal synchrony during conjunctive than non-conjunctive (feature) search. Whether this difference also reflects greater information integration, rather than just differences in cognitive strategy (e.g., top-down versus bottom-up control of attention), or task difficulty is uncertain. Here, we examine the first possibility by parametrically varying the number of integrated sources from one to three and measuring phase-locking values (PLV) of frontal-parietal EEG electrode signals, as indicators of synchrony. Linear regressions, under hierarchical false-discovery rate control, indicated significant positive slopes for number of sources on PLV in the 30–38 Hz, 175–250 ms post-stimulus frequency-time band for pairs in the sagittal plane (i.e., F3-P3, Fz-Pz, F4-P4), after equating conditions for behavioural performance (to exclude effects due to task difficulty). No such effects were observed for pairs in the transverse plane (i.e., F3-F4, C3-C4, P3-P4). These results provide support for the idea that anterior-posterior phase-locking in the lower gamma-band mediates integration of visual information. They also provide a potential window into cognitive development, seen as developing the capacity to integrate more sources of information.

## Introduction

The capacity to integrate multiple sources of information is a prerequisite for complex cognitive behaviour. From vision [1] to reasoning [2], a wide variety of experimental paradigms have been employed to elucidate the processes that underlie information integration. In this regard, visual search tasks have been particularly fruitful at both behavioural and neural levels, because they involve relatively simple procedures and modifications that span both perceptual and cognitive (attentional) domains while being amenable to the constraints of neuroimaging.

To contrast unintegrated versus integrated information in a visual search task, participants are typically required to find a target object that is uniquely identifiable by a single feature (feature search) versus a tuple of features (conjunctive search) that are selected from, say, the colour dimension versus the colour and orientation dimensions (see [3] for a review). At the behavioural level, feature search is often relatively fast, accurate and efficient (i.e., less adversely affected by the number of items in the search display), whereas conjunctive search is often relatively slow, inaccurate and inefficient (i.e., more adversely affected by the number of items in the search display) [4]. This difference is often characterized as bottom-up (stimulus-driven) versus top-down (context-driven) control of attention (e.g., [4], [5]).

Inspired by work on primitive feature binding in early vision (see [1] for a review), a popular framework for modeling information integration at the neural level is temporal synchrony (see [6]). For instance, two sources of information (say, a colour and an orientation) may be integrated (or bound to a common object) by the phases of their respective carrier signals (phase-synchrony): e.g., red and vertical are bound to one object because the units (neurons) encoding these features oscillate in-phase with respect to each other (due to their common source location), but out of phase with respect to the colours and orientations associated with objects at other locations in the field of view. At the neuronal level, overlapping receptive fields, both from bottom-up and top-down influences, can drive temporally correlated activation between cell assemblies for the same complex object that mutually enhance/suppress activity from cells firing in/out of phase via recurrent excitatory/inhibitory connections (see [7]–[9]). Computationally, each phase can act like a unique tag that identifies each collection of components (assembly) as a particular whole from which the components can be subsequently retrieved.

A number of neural network architectures employ synchrony to model visual (e.g., [10]), and cognitive processes more generally (e.g., [11]–[13]), as a way of building representations of complex entities out of the representations of their constituents. Such computational principles suggest that temporal synchrony may also mediate information integration for higher cognitive processes, not just the binding of sensory features in early vision. For this reason, we use the more general term integration, rather than binding.

Indeed, evidence of temporal synchrony associated with attentional control was found in monkeys (with implanted electrodes) performing a visual search task [14]. This study contrasted frontal and parietal activity for conjunctive (target uniquely identifiable by two features) versus feature (target uniquely identifiable by one feature) search. Buschman and Miller [14] observed greater frontal-parietal synchrony for conjunctive than feature search in a lower frequency band (22–36 Hz), but greater synchrony for feature than conjunctive search in a higher band (38–54 Hz). Subsequent work on humans using scalp electroencephalography (EEG) also reported greater anterior-posterior synchrony for the conjunctive than feature condition in the same lower frequency band [15], using EEG phase-locking values (PLV) as measures of synchrony [16].

These studies [14], [15], however, were primarily designed to assess the differences in top-down and bottom-up control of attention–the single feature condition in these studies also corresponds to the so-called popout condition (i.e., where all non-targets have the same features not shared with the target), which is generally faster and more efficient than when non-targets are also distinct from each other (i.e., the distractor-distractor similarity effect) [17], [18]. Consistent with the bottom-up versus top-down distinction, Buschman and Miller [14] also found that activity in parietal cortex preceded frontal cortex in the popout condition, but the reverse order of activation in the slower conjunctive search condition.

For the purpose of identifying information integration-related effects, there are several difficulties associated with contrasts of conjunctive versus popout search. Firstly, changes in (EEG) measures of synchrony may also be associated with general task difficulty rather than a putative integration process, since conjunctive search is generally slower and more error prone than popout search [4]. Secondly, if conjunctive and popout search employ two different types of cognitive processes (i.e., top-down versus bottom-up control), then observed differences in synchrony may reflect more general differences in control processes instead of differences that are specific to integration.

Coupled with these specific challenges is a more general issue associated with the apparent transient and frequency-dependent nature of feature integration: instantaneous frequency-time measures of synchrony (e.g., PLV) incur a serious multiple comparisons problem–in general, the likelihood of false-positives increases when more contingencies are considered, and hence more tests are conducted. In our context, that integration effects may be contingent on specific frequencies and time delays necessitates more tests (e.g., one for each frequency and time point combination) and greater likelihood of observing differences due to chance only.

In the present study, we address these issues using a parametric design by measuring EEG synchrony for targets and non-targets that are both uniquely identifiable along one, two, or three visual feature dimensions (see Methods for details). In this case, the one-dimensional condition is not equivalent to popout, because the non-targets are also unique. Since the similarity between non-targets (distractors) is low, search difficulty is more likely to be greater, and comparable with the other conditions. Moreover, identifying a monotonic increase in synchrony is more likely to be directly related to integration, than differences in process type (see [19] for the advantages of parametric over subtractive/additive designs). The multiple comparisons problem is also addressed with a method called hierarchical false discovery rate (hFDR [20]), which we recently adapted to the analysis of PLV in EEG data [21]). In light of our earlier result [15], we hypothesize a significant increase in synchrony (PLV) between frontal and parietal EEG electrodes as a function of the number of feature dimensions that uniquely identify the target in a 22–34 Hz frequency band around 160–480 ms into the search task (i.e., after the presentation of the search display).

## Methods

All procedures were approved by the National Institute of Advanced Industrial Science and Technology (AIST) Safety and Ethics committee, and conducted after receiving written informed consent from the participants.

### Participants

Seventeen Japanese university students (3 female, 2 left-handed) participated in the experiment, aged 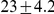 years (mean 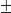 stddev). Data from two additional participants was excluded from subsequent analysis due to an insufficient correct response rate (less than 50%) in one case, and excessive noise in the raw EEG signal (particularly channels T3, T4, T5, and T6, suggesting electromyogram confounds) in the other case. (Muscle movement artifacts are usually removed by low-pass filtering, but low-pass filtering conflicts with PLV analysis in the gamma-band, so all data for this participant were removed.) Participants had normal, or corrected-to-normal vision. They were paid to participate in the experiment.

### Apparatus and stimuli

Stimuli were presented using a standard desktop computer (PC) and a 21-inch LCD placed about 57 cm from the participant (so that 1 cm equals about 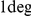 field of view). Screen resolution was 1600×1200 pixels and the refresh rate was 60 Hz. The field of view in the horizontal and vertical directions was approximately 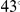 and 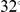, respectively. Stimuli were rectangular bars on a gray background, subtending 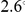 in length and 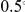 in width. The angular difference between bars (centre-to-centre) was approximately 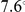. Each bar was constructed with a (colour, orientation, frequency) feature triple. Each feature dimension had four possible values. Colour was either red, green, blue, or yellow; orientation was either 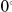, 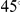, 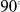, or 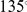 degrees from horizontal; and frequency referred to either 0, 1, 2, or 3 gaps (square holes) at regular intervals in the rectangular bar (filled by the background colour). The display was divided into four equal quadrants by invisible horizontal and vertical centerlines. Each quadrant contained one stimulus item, jittered about its center so that the location of the target was clearly identifiable by the containing quadrant. Electrical potentials were collected using a digital electroencephalograph system (Nihon Kohden Neurofax EEG-1100) with an Ag/AgCl electrode cap. The stimulus presentation (PC) and EEG acquisition systems were synchronized at the presentation of the search display (see below) of each trial: after updating the display using the openGL *double buffering* facility, a signal was sent to the EEG acquisition system via the serial port (PC) marking the start of search.

### Conditions

**Figure 1:**
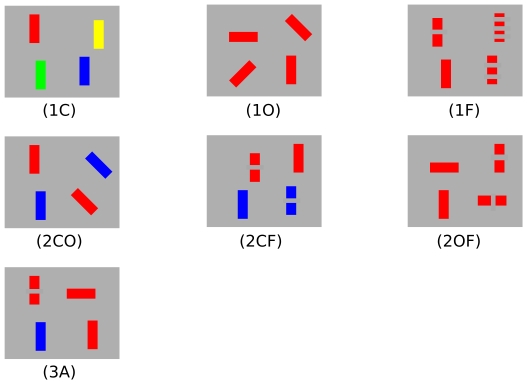
Example displays. Example display sets for each arity-dimension condition. In each case, the target is the (red, 90, 0-gap) object.

There were three search display arity conditions: unary (1), binary (2), and ternary (3), where the target and non-targets in each display set were uniquely identifiable by one, two, and three features (respectively). There are two (one) irrelevant dimensions in the unary (binary) condition for which all target and non-target objects in the display set share the same feature on that dimension. An example display set for the binary condition is indicated by the following four (colour, orientation, frequency) feature triples: (red, 90, 0-gap)–target, (red 45, 0-gap), (blue, 90, 0-gap), and (blue, 45, 0-gap)–non-targets, where the colour and orientation dimensions uniquely identify each triple (i.e., object) and frequency is the irrelevant dimension, in this example. Examples of each of the seven arity-dimension conditions is shown in Figure 1. For the unary and binary conditions, there were three dimension conditions associated with the colour (C), orientation (O) and frequency (F) feature dimension(s) that uniquely identified the target. In the ternary condition, all (A) features are needed to identify the target. In total, there were seven conditions, which we label 1C, 1O, 1F, 2CO, 2CF, 2OF, and 3A.

### Procedure

**Figure 2:**
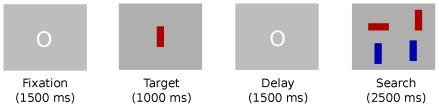
Example trial. Each trial consists of fixation (1500 ms), target (1000 ms), delay (1000 ms), and search display (2500 ms) periods.

Each trial consisted of four periods in the following order: (1) fixation (1500 ms), when participants focused on a small white ring placed at the center of the screen; (2) target display (1000 ms), with the target positioned at the screen center; (3) delay (1000 ms), with just the background colour; and (4) search display (2500 ms, or until a key was pressed, whichever came first), when the four bars were displayed, one for each quadrant. During the search display period, participants were required to identify the target location within the 2500 ms time limit by pressing the key corresponding to the quadrant where the target was located. Speed and accuracy of response were stressed. Participants pressed either key ‘a’ (upper left), or ‘z’ (lower left) with their left hand; or ‘k’ (upper right), or ‘m’ (lower right) with their preferred hand to identify quadrants. The assignment of stimulus items and responses to quadrants was randomized and counterbalanced across trials (respectively). Trial timing and example search displays are shown in Figure 2.

Each person participated in two sessions. Each session consisted of 27 ( = 3 [arity]×3 [dimension]×3 [repetition]) blocks of trials. For the ternary arity condition (3A), all three dimensions are needed to identify the target, hence the arity-dimension combinations ternary-colour, ternary-orientation, and ternary-frequency are not distinguished. Each block was preceded by a prompt screen (10 s), indicating the imminent start of the next block. There were 10 trials per block. Hence, each participant received 540 experiment trials ( = 2 sessions×27 blocks×10 trials). Block and trial types were randomized. Prior to the first session there was also a short practice session (about 3 min) to familiarize the participants with the display stimuli and ensure they understood the procedure. Response keys and times were recorded. Pressing an incorrect key, or failure to respond within the maximum allotted time was regarded as an error.

Electroencephalograms (EEG) were measured from the following 19 electrode sites of the International 10–20 system: Fp1, Fp2, F7, F3, Fz, F4, F8, T3, C3, Cz, C4, T4, T5, P3, Pz, P4, T6, O1, and O2, with AFz as the ground electrode. Reference potentials were recorded from electrodes A1 and A2, one attached to each earlobe. A vertical electrooculogram (EOG) was recorded using electrodes placed above and below the right eye, and a horizontal EOG was recorded from the outer left and right canthi to monitor possible artifacts due to eye movements. Electrodes were attached with gel to reduce impedance to below 5 k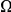. EEG and EOG were digitized at a rate of 1000 Hz, and were band-pass filtered at 0.032 Hz and 300 Hz. The experiment was conducted inside an electrically shielded room.

Before commencing the experiment sessions, participants received general instructions regarding the experiment and EEG/EOG procedure. After attaching the head cap and electrodes, participants were given specific instructions regarding the task, followed by a short demonstration. The total time required for practice and experiment sessions was about 1.5 hr.

### Analysis

Analyses of variance (ANOVAs) were conducted on response errors and times. Analysis of response time was done after removing error and outlier trials (2% of trials), which were determined by the modified recursive method [22] on error-free trials. Data were entered into 1-way (arity) repeated measures ANOVAs to infer significant effects. Tukey's HSD analysis for seven levels was conducted to assess differences between means for the seven arity-dimension conditions. For response errors, where error rates are bounded by 0 and 1, an arcsine transform (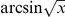) was applied to error rates to stabilize variances before conducting analysis (see [23]).

EEG data were re-referenced offline to the mean of earlobe potentials A1 and A2. A data window was set at −200 ms to 1000 ms relative to search display onset. Trials containing EEG artifacts (e.g., eye blinks), approximately 25% of trials, identified by visual inspection, or response errors were excluded from further analysis. Independent components analysis (ICA), as implemented in EEGLab [24], was used to further remove eye movement related components, and PLV analysis was conducted on the remaining ICA components.

Phase-locking values (*PLV* [16]) were used as measures of synchrony. PLVs were computed from stimulus-locked EEG data for each trial as measures of synchronization between brain regions. The phase *ϕ* 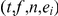 at time (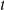), frequency (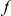), trial (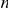) and electrode (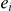) was computed by first convolving the data with a complex Morlet wavelet, defined as:
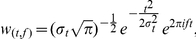
where 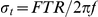. Following [16], we set 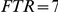. For the purpose of linear regression analysis, 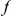 ranged from 10 Hz to 58 Hz at intervals of 2 Hz. 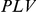 for electrode pair 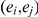 was computed as:
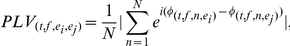
where 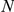 is the number of trials. Thus, a PLV of 1 indicates a constant phase difference across trials at a particular time point and frequency component. Conversely, a randomly varying phase difference across trials has a PLV of 0. PLVs were normalized [25]. The normalized values, 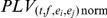, were computed for each 1 ms time point as:
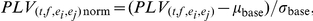
where 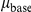 and 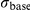 are the mean and standard deviation PLV over a baseline period from 200 ms to 0 ms prior to stimulus onset. Hence, normalized PLVs are no longer bounded between 0 and 1. PLVs were downsampled by taking every fourth time point.

For the purpose of identifying significant PLV-arity slopes for electrode pairs at specific frequencies and times, the following procedure was performed. Frequency was divided into 12 contiguous 4 Hz bands from 10–58 Hz. Time was divided into 22 contiguous 25 ms bands from 50–600 ms (post-stimulus–search display–onset). The 0–50 ms time bands were omitted from analysis, because it takes approximately 50 ms for information to reach the primary visual cortex [26], [27]. The slopes from linear regressions of arity onto PLV were computed from the mean PLVs for each frequency-time band and electrode pair combination, and their significances were corrected for multiple comparisons using the hierarchical false discovery rate (hFDR) method [20] (see next). All 171 electrode pairs were considered in the initial stage of analysis, but hFDR failed to detect any significant effects. Pairs F3-P3, Fz-Pz, and F4-P4 were considered as the primary region (electrode pairs) of interest following our earlier study [15], which were used to approximate the ipsilateralized positioning of frontal and parietal electrodes in the [14] study. Other pairs were also considered in subsequent analyses (see below). Although the false discovery rate (FDR) method first proposed in [28] is widely used for controlling false positives in neuroimaging, we found FDR to be too conservative for the analysis of PLV in EEG data. Instead, hFDR has proven to be more sensitive without unduly increasing the rate of false discoveries [21].

For the purpose of applying hFDR analysis, all hypotheses were organized into a three-level family-hypothesis tree hierarchy, where the first level is associated with frequency, the second level with time, and the third level with electrode pair. (Analogous to a parent-child family tree, each hierarchically organized hypothesis belongs to one and only one family. The structure is recursive, so a hypothesis that is a member of one family may also represent a family of hypotheses at the next level below.) The first level constitutes a single family of 12 frequency-dependent hypotheses: i.e., for each frequency band there is one (summary) null hypothesis saying that the slope computed from the mean of the PLVs for all frequency, time and electrode pair combinations associated with that frequency band is not significantly different from zero. The second level constitutes 12 families (i.e., one for each frequency band) of 24 frequency-time-dependent hypotheses each: i.e., for each time band (within each frequency band) there is one (summary) null hypothesis saying that the slope computed from the mean of the PLVs for all frequency, time and electrode pair combinations associated with that frequency-time band is not significantly different from zero. Finally, the third level constitutes 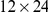 families (i.e., one for each frequency-time band) of electrode pair-dependent hypotheses each, where the null hypothesis says that the PLV-arity slope associated with this frequency, time, electrode pair combination is not significantly different from zero. The actual pairs analyzed are specified in the next section.

The hFDR procedure for determining significance basically proceeds recursively from the first to the last level. In our case, that means the following. At the first level, a *p*-value is computed for each of the frequency-dependent null hypotheses, and the resulting 12 *p*-values are thresholded by the FDR procedure for that family. The null hypotheses corresponding to the *p*-values that survived the FDR threshold are rejected. Hypotheses not rejected are excluded from further analysis, including their member hypotheses when considered for the next level of analysis. For each rejected hypothesis (corresponding to a frequency band that survived cutoff), the hFDR procedure is applied recursively to their member hypotheses: i.e., when each rejected hypothesis is considered as a family of hypotheses at the next level of hFDR analysis, which means the 24 frequency-time-dependent hypotheses. In our case, this procedure terminates at either: (1) the second level, when we are concerned with identifying significant frequency-time bands for the regions (electrode pairs) of interest (see next); or (2) the third level, when we are also concerned with identifying specific pairs within the region of interest. A hFDR upper bound can also be computed with this method, which estimates the total false discovery rate incurred due to additional testing associated with the construction of hypothesis families. The FDR threshold for each level of hFDR testing was set at 0.04 to maintain the hFDR bound at about 5%. See [21] for details on hFDR analysis and computation of hFDR bound as applied to EEG phase-locking data.

### Contrasts and regions (electrode pairs) of interest

For the purpose of identifying PLV effects beyond those due to task difficulty, we analyzed the data obtained from all conditions (full data set), and a subset of the data that included only those conditions regarded as “equivalent” in terms of difficulty (reduced data set, see below).

PLV analysis was also refined to specific pairs of frontal, central, and parietal electrodes. Three-level hFDR analysis, which included all 171 electrode pairs at each frequency-time band, and all combinations of a subset of frontal (F3, Fz, F4), central (C3, Cz, C4), and parietal (P3, Pz, P4) electrodes (i.e., 72 pairs), did not reveal any significant slopes (at an FDR of 5%) for either data set. For the purpose of increasing the sensitivity of our analysis, we considered the three pairs (F3-P3, Fz-Pz, and F4-P4) as in our previous study as a single summary electrode pair. That is, a two-level hFDR with frequency at the first level and time at the second level was conducted on a summary frontal-parietal electrode pair: i.e., the mean of the three frontal-parietal pairs in our primary region of interest.

Although our analysis does not identify the sources of synchrony, it can be used to differentiate synchrony in the sagittal planes (i.e., anterior-posterior connections) versus transverse planes (i.e., left-right hemisphere connections). Accordingly, follow up analyses considered six pairs of electrodes at the third level: that is, three sagittal pair: F3-P3, Fz-Pz, F4-P4; and three transverse pairs: F3-F4, C3-C4, and P3-P4 (see Text S1).

## Results

### Behaviour

ANOVAs revealed a significant effect of arity on response error rate, 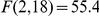, 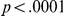, and response time, 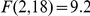, 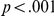. Post hoc analysis revealed significant differences between mean ternary and unary, and ternary and binary error rates (respectively, 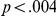 and 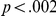), and response times (respectively, 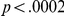 and 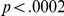). Differences between unary and binary means were not significant. Mean error rates and response times for each arity condition are shown in Table 1.

**Table 1:** Response by arity condition.

Condition	Unary	Binary	Ternary
Error rate	.03 (.007)	.03 (.008)	.05 (.010)
Response time	724 (39)	706 (42)	788 (43)

Mean (standard error) response error rates and times (ms) for each arity condition.

For the purpose of assessing arity effects that are independent of task difficulty (as measured by error rates and response times), analysis of arity-dimension mean was also conducted. For this analysis, we are interested in the unary-dimension and binary-dimension conditions where error rates and response times are not significantly better than the ternary condition. The logic is that any significant differences observed in subsequent PLV analysis cannot be attributed simply to increased task difficulty. For the unary-dimension conditions, the error rate for 1O (.05) and 1F (.03) conditions were not significantly different from 3A (.05), 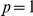 and 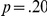 (respectively), and the response time for 1O (894 ms) was greater than for 3A (788 ms), which was not significantly different from 1F (763 ms). For the binary-dimension conditions, only performance in the 2OF was not significantly better than 3A–the difference between error rates for 2OF (.04) and 3A (.05) was not significant (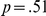), and the response time for 2OF (795 ms) was greater than for 3A (788 ms). These conditions (1O/1F–unary, 2OF–binary, and 3A–ternary) were used in the second stage of PLV analysis (next section) to assess arity effects on EEG synchrony independently of task difficulty. Mean error rates and times for each arity-dimension condition are shown in Table 2.

**Table 2:** Response by arity-dimension condition.

Condition	1C	1O	1F	2CO	2CF	2OF	3A
Error rate	.01 (.006)	.05 (.011)	.03 (.001)	.03 (.011)	.02 (005)	.04 (.011)	.05 (.01)
Response time	525 (36)	894 (39)	763 (45)	694 (37)	645 (46)	795 (42)	788 (43)

Mean (standard error) response error rates and times (ms) for each arity-dimension condition.

To confirm that the 1O (unary-orientation) condition was indeed at least as difficult as the 3A (ternary) condition, the behavioural component of the experiment was repeated with the original stimuli for one session, and with 0.7 magnification of the original stimuli for another session. The response errors and times for this second experiment confirmed that visual search in the 1O condition was at least as difficult as for the 3A condition (see Text S2).

### Phase-locking (PLV)

**Figure 3:**
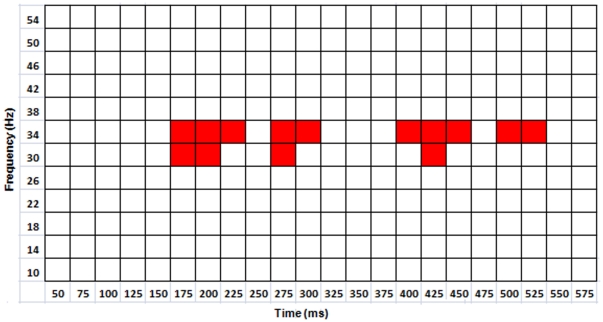
PLV regressions for full data set. Each red square indicates a significant positive slope (hFDR corrected, 5% hFDR bound) for PLV as a function of arity from the mean of F3-P3, Fz-Pz, and F4-P4 electrode pairs for the indicated frequency-time band.

**Figure 4:**
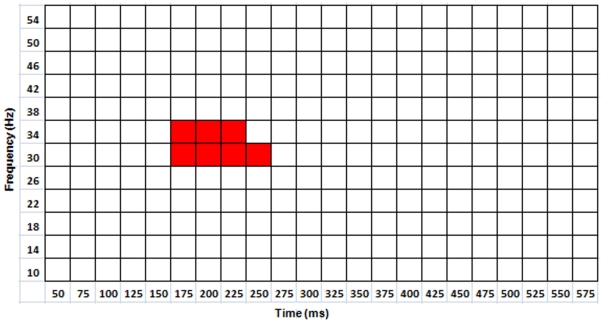
PLV regressions for reduced data set. Each red square indicates a significant positive slope (hFDR corrected, 5% hFDR bound) for PLV as a function of arity from the mean of F3-P3, Fz-Pz, and F4-P4 electrode pairs for the indicated frequency-time band.

**Figure 5:**
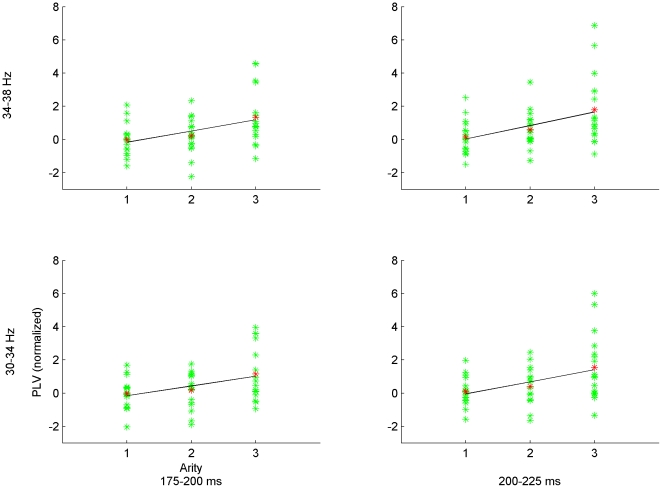
PLV-arity plots for full data set. Plot of PLV at each arity for each participant (green) and their mean (red) for significant frequency-time bands from the mean of F3-P3, Fz-Pz, and F4-P4 electrode pairs. Line indicates the regression slope.

**Figure 6:**
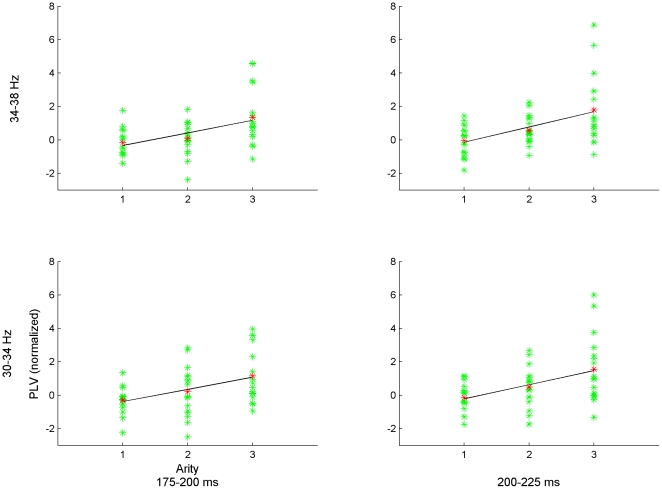
PLV-arity plots for reduced data set. Plot of PLV at each arity for each participant (green) and their mean (red) for significant frequency-time bands from the mean of F3-P3, Fz-Pz, and F4-P4 electrode pairs. Line indicates the regression slope.

For the full data set (i.e., involving all seven arity-dimension conditions), regression analysis with two-level hFDR control revealed significant positive slopes in the 30–38 Hz frequency band, within four intervals: 175–250 ms, 275–325 ms, 400–475 ms, and 500–550 ms post-stimulus onset, for the mean of the three frontal-parietal electrode pairs (see Figure 3). For the reduced data set (i.e., including only those unary/binary-dimension conditions where error rate and response time were not significantly higher in the ternary condition), regression analysis revealed a single 30–38 Hz band at 175–275 ms post-stimulus onset (see Figure 4). The FDR threshold for each level of hFDR was set at 0.04 for both data sets. The estimated hFDR bound for the full data set was 0.054, and for the reduced data set was 0.04. Regression slopes were plotted along with the (mean) PLVs at each arity and participant for several significant frequency-time bands both for full and reduced data sets (see Figures 5 and 6, respectively). Results from additional analyses are provided in Text S1.

## Discussion

Regressions of arity onto PLV, controlled for multiple comparisons (using hFDR) revealed positive slopes in a frequency-time band (30–38 Hz at 175–250 ms post-stimulus onset) that is consistent with our previous study [15]. The monotonic increase in PLV with arity under conditions of equivalent behavioural performance suggests that this band is associated with integrating visual features, not general task difficulty. Moreover, this effect is unlikely to be due to differences in cognitive strategy (e.g., top-down versus bottom-up control) since both binary and ternary conditions involve conjunctive search.

Although our analysis does not identify the sources of PLV synchrony, likely locations are frontal and parietal cortices, given is its prominence in other studies (in particular [14], examining local field potentials, which can identify locations more accurately than EEG). Volume conduction makes source identification difficult, since the signal from each generator disperses over the scalp. We have attempted to mitigate this problem by testing three other electrode pairs (F3-F4, C3-C4, and P3-P4, referred to as the transverse pairs) that are comparable to the significant pairs (F3-P3, Fz-Pz, and F4-P4, referred to as the sagittal pairs) as identified by hFDR under the assumption that synchrony effects were due volume conduction: since the electrodes in each pair are approximately equidistant, significant PLV effects should also be observed with the transverse pairs (see Text S1). Even under the less conservative cutoff of 5%, uncorrected, there were relatively few occurrences of transverse (than sagittal) pairs that survived this criterion within the 30–38 Hz at 175–250 ms band identified as significant from hFDR analysis: respectively, 0 versus 13 occurrences (full data set), and 3 versus 14 occurrences (reduced data set). Our results suggest that synchrony was primarily due to anterior-posterior connectivity. Volume conductivity alone does not discriminate pairs, since electrode separation within each pair is roughly the same.

The use of the regression statistic in the hFDR context is a novel application of hFDR method for controlling false positives. As pointed out in [20], hFDR is a general framework for FDR control, and hence not limited to the commonly used *t*-statistic. For our purposes, regression is particularly appropriate as we are interested in the effect of arity on phase-locking, rather than the significance of individual means. In general, hFDR is more powerful than FDR. In particular, FDR did not reveal any significant phase-locking effects for the present study.

Although the identified frequency-time band is consistent with our previous study, we did not observe a significant difference between the individual binary and unary arity means, unlike in our previous work. One possible reason for this difference concerns the specific nature of the unary condition. As mentioned in the Introduction, last paragraph, the two unary conditions differ in the construction of non-targets, which were identical in the previous study, but different in the current one. Hence, the unary and binary conditions are more comparable in the current study than the previous one, and so PLVs for the unary and binary conditions here are likely to need more data to distinguish at the same level of significance.

This relationship between arity (as a degree of information integration) and PLV that we have identified has potential implications for cognitive development, and cognitive processing more broadly. A general *category theory* [29] treatment of information integration was provided in [30] (see also [31], [32]) to explain common differences between younger and older children's capacity (relative to five years of age) for inference across a variety of reasoning tasks. Essentially, the difficult condition in each task involved computing a binary fibred (co)product, i.e., integrating two sources of information, whereas the easy condition did not–equivalent to a unary fibred (co)product, i.e., only one source of information. Younger children were generally successful only on easy versions on each task, hence their capacity is limited to a single information source. In contrast, older children were also successful on the difficult versions of each task, hence they have the capacity to integrate two sources of information (see [30] and the cited empirical studies therein for details).

In the current context of visual search, one, two and three dimensional feature search involves computing a unary, binary, and ternary fibred product (respectively), where arity is the number of product arguments (see Text S3). Although display objects in all conditions always had three features (i.e., a colour, orientation, and frequency), one or two of these features were constant across (i.e., shared by) all objects in the search display set for the binary and unary arity conditions (respectively). In line with the category theory explanation for task difficulty [30], the arity of a fibred product involving a constant dimension reduces (i.e., is isomorphic) to a fibred product of a lower arity. Search over a constant dimension (where every display object has the same feature on that dimension) involves a lower arity fibred product: Consider a fibred product as the Cartesian product of each feature-location map constrained by object location. Arity corresponds to the number of feature-location maps needed to identify an object. Hence, for unary (binary) conditions, where objects are uniquely identifiable on one (two) feature dimension(s), search involves a unary (binary) fibred product (see Text S3 for details).

Further support for this category-theoretic treatment of information integration comes from recent evidence indicating that younger children (4-year-olds) have difficulty on visual memory tasks involving feature conjunctions [33], but no such difficulty for items uniquely identifiable by a single feature. This situation corresponds to a binary versus unary fibred product, though the authors of [33] emphasized the differences in terms of retrieval not encoding deficits. In the context of the current study, we suggest that developmental differences may also be revealed in the form of significantly greater frontal-parietal synchrony in the lower gamma-band for older than younger children.

Whether (frontal-parietal) synchrony underlies information integration for higher cognition (e.g, reasoning) remains to be investigated. For visual search, location is usually the basis for feature integration, so parietal involvement is natural given its role in spatial attention [34]. Reasoning, though, generally depends on item relationships that transcend spatial contexts, and neuroimaging research has typically focussed on the role of prefrontal cortex (e.g., [35], [36]). However, one conception of working memory says that complex cognition (including reasoning) requires dynamic binding of items to “places in a cognitive coordinate system” [37]. If parietal cortex is home to such coordinate systems, then we can expect frontal-parietal synchrony to also mediate information integration for higher-level cognition.

## Supporting Information

Text S1 **PLV regressions for sagittal and transverse electrode pairs.**
(PDF)

Text S2 **Experiment replication for behavioural responses.**
(PDF)

Text S3 **Visual search and fibred products.**
(PDF)
