# Supplementary material for: Visual Feature Integration Indicated by pHase-Locked Frontal-Parietal EEG Signals
Source: PLoS One. 2012 Mar 9;7(3):e32502. doi: 10.1371/journal.pone.0032502 (PMC3302878; doi:10.1371/journal.pone.0032502)
Supplement: Text S1 — PLV regressions for sagittal and transverse electrode pairs. (PDF) [file pone.0032502.s001.pdf]

Text S1

To differentiate the directionality of synchrony six electrode pairs were included for analysis. They are the three pairs from our primary region of interest: F3-P3, Fz-Pz, F4-P4 (sagittal pairs), and three pairs in the transverse direction for the same region: i.e., F3-F4, C3-C4, P3-P4 (transverse pairs). Regression analysis of arity onto PLV was conducted without hFDR correction at a cutoff of 5%. For the significant frequency-time bands identified in the primary hFDR analysis (i.e., 30–38 Hz at 175–250 ms), synchrony was predominantly with the sagittal pairs for both the full (Figure 1) and reduced data sets (Figure 2). For the frequency-time bands identified as significant by hFDR analysis that are common to both full and reduced data sets (i.e., 33–34 Hz at 175–225 ms and 34–38 Hz at 175–250 ms) there were 0 occurrences of transverse pairs that survived cutoff compared to 13 occurrences of sagittal pairs for the full data set, and (respectively) 3 compared to 14 occurrences for the reduced data set.

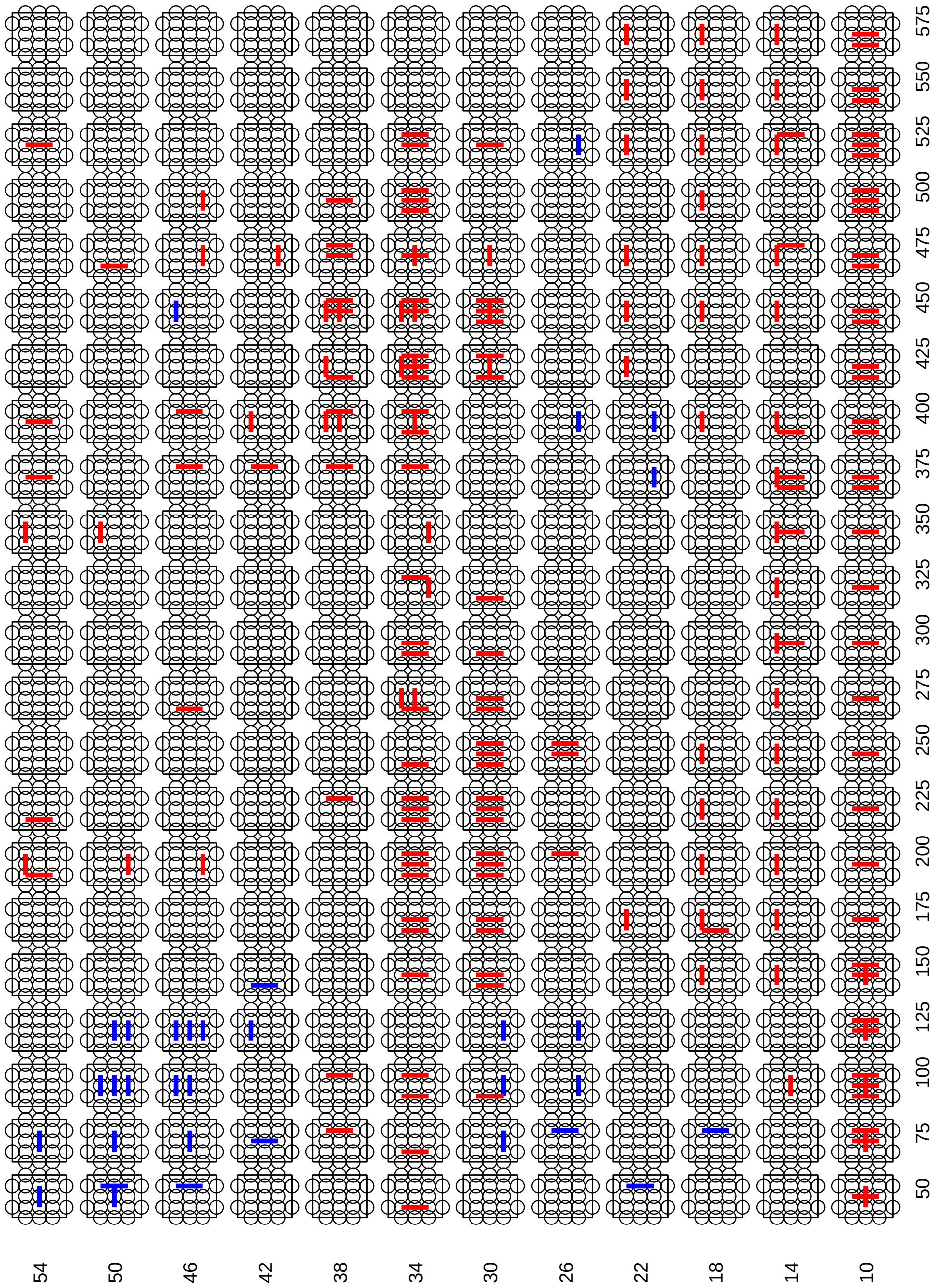

Figure 1. PLV slopes by directionality for the reduced data set (uncorrected). Each red (blue) line indicates a positive (negative) PLV-arity slope for sagittal (F3-P3, Fz-Pz, F4-P4) and transverse (F3-F4, C3-C4, P3-P4) pairs, at a 5% cutoff, uncorrected, at the indicated frequency-time bands.

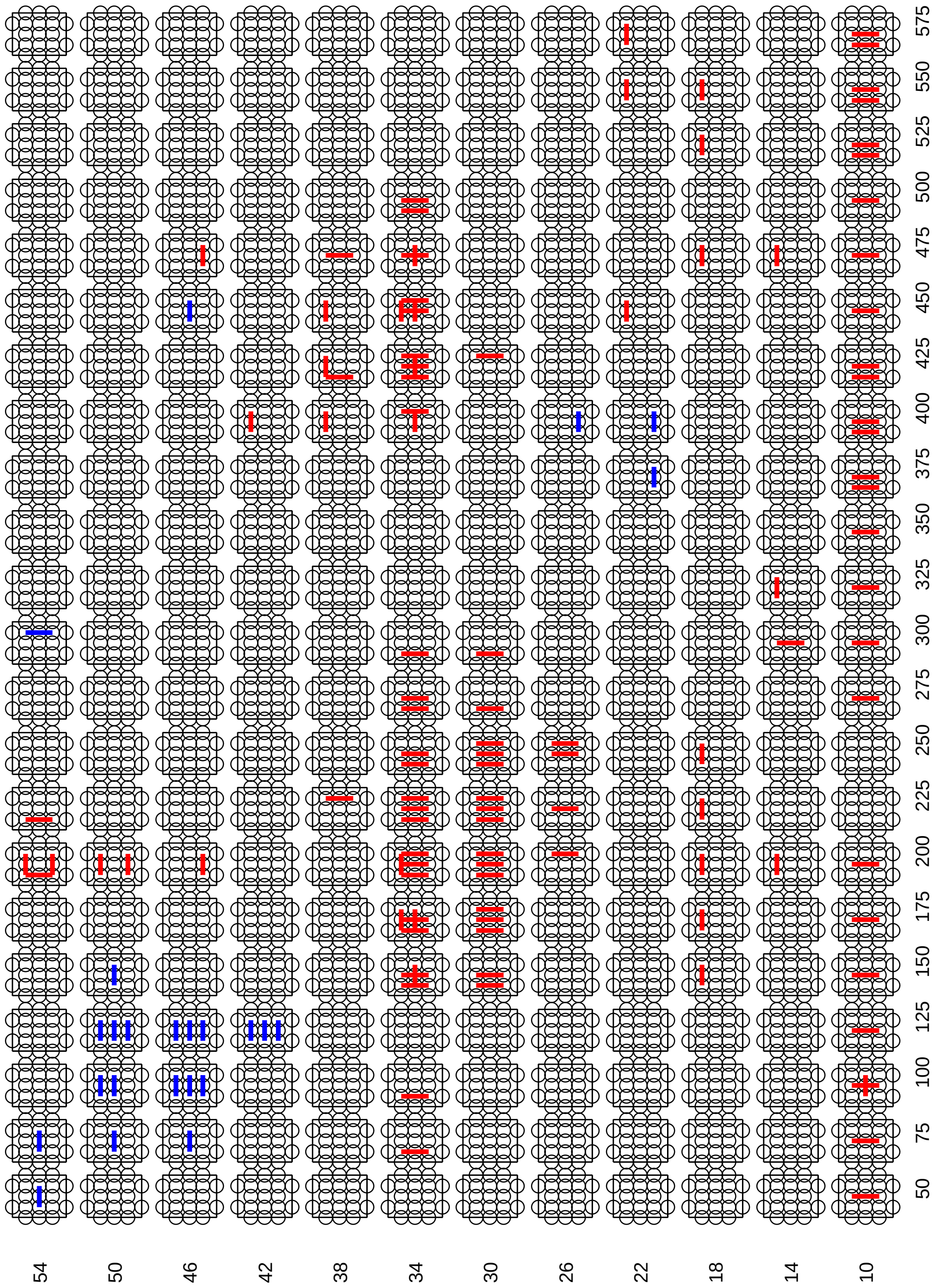

Figure 2. PLV slopes by directionality for the reduced data set (uncorrected). Each red (blue) line indicates a positive (negative) PLV-arity slope for sagittal (F3-P3, Fz-Pz, F4-P4) and transverse (F3-F4, C3-C4, P3-P4) pairs, at a 5% cutoff, uncorrected, at the indicated frequency-time bands.
