# Supplementary material for: Visual Feature Integration Indicated by pHase-Locked Frontal-Parietal EEG Signals
Source: PLoS One. 2012 Mar 9;7(3):e32502. doi: 10.1371/journal.pone.0032502 (PMC3302878; doi:10.1371/journal.pone.0032502)
Supplement: Text S2 — Experiment replication for behavioural responses. (PDF) [file pone.0032502.s002.pdf]

Text S2

The behavioural component of the experiment described in the main text was repeated. We also included an additional set of stimuli (i.e., the original stimuli scaled down to about three-quarters of their original size) to investigate the possibility that interference between stimulus orientation features caused the increase in search difficulty. Only those aspects of the new experiment that differed from the previous one are detailed here.

Methods

All procedures were approved by the National Institute of Advanced Industrial Science and Technology (AIST) Safety and Ethics committee, and conducted after receiving written informed consent from the participants.

Participants

Ten Japanese university students (5 female, all right-handed) participated in the experiment, aged 23.7 ± 4.2 years (mean $\pm$ stddev).

Stimuli

Two types of stimuli were presented to participants, labelled "large" (which were identical to the stimuli used in the previous experiment), and "small" (which were the large stimuli magnified by a factor of 0.7).

Conditions

All participants did two sessions: one session with the large set of stimuli, and one session with the small set of stimuli. (The conditions for each session were otherwise the same as the previous experiment.)

Procedure

The participants were divided into two groups of five. One group did the session with the large stimuli first, followed by the session with the small stimuli. Session order was reversed for the other group.

Results and discussion

For response time, an ANOVA revealed a significant main effect of arity $F(2, 18) = 14.0, p < .001$, but not stimulus size $F(1, 9) = 0.99, p = .35$. The interaction between arity and stimulus size was not significant ($p > .63$). For large stimuli only (i.e., the same stimuli used in the previous experiment), the mean response times for each arity condition were 664 ms (unary), 656 ms (binary), 725 ms (ternary). For conditions 1O, 1F, 2OF, and 3A (regarded as "equivalent" in difficulty, in the previous experiment), the mean response times for large stimuli were 812 ms, 716 ms, 736 ms, and 725 ms(respectively). Post hoc analysis (Tukey's HSD) revealed a significant difference between 1O and 3A ($p < .001$), but not between 1F and 3A ($p = .95$), nor between 2OF and 3A ($p = .92$).

For response error, an ANOVA did not revealed a significant main effect of arity ($p = .76$), nor stimulus size ($p = .52$). For large stimuli only, the mean error rates were 0.032 (unary), 0.035 (binary), 0.041 (ternary). For conditions 1O, 1F, 2OF, and 3A, the mean error rates for large stimuli were 0.020, 0.062, 0.053, and 0.041 (respectively). Post hoc analysis revealed no significant difference between 1O and 3A ($p = .69$), 1F and 3A ($p = .96$), nor 2OF and 3A ($p = .22$). Mean response times and error rates for each condition are shown in Figure 1.

Consistent with the first experiment, conditions 1O, 1F, and 2OF were not significantly easier than 3A. For the most part, mean response times and error rates were about the same or higher than 3A. Thus, we conclude that changes in PLV were not solely due to task difficulty.

The response time for the unary-orientation (1O) condition, though, was significantly longer than than the ternary condition (3A), and the use of smaller stimuli (to reduce between item interference) did not make search easier. The greater search time for orientation is surprising, given that orientation is generally regarded as a feature that affords efficient search in the presence of homogeneous distractors [1]. However, in the presence of heterogeneous distractors, researchers have also reported that search involving a unique orientation can also be distinctly inefficient [2]. Wolfe et. al [2] also reported that search can be made more efficient when distractor orientation features can be classified into a single group—essentially, distractors become homogeneous at the categorization level. In our case, though, distractors we uniformly different from each other, precluding such a strategy. Our stimuli also included additional orientation information in the form of gaps to indicate the frequency feature, which may have made the orientation condition more difficult.

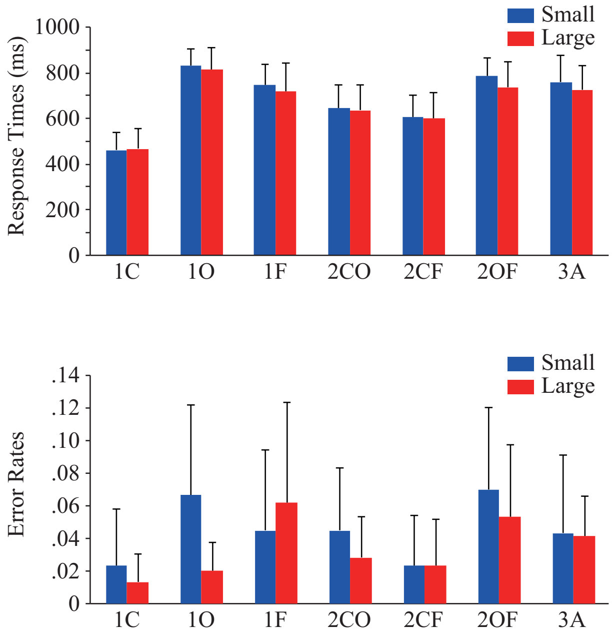

Figure 1. Mean response times and error rates. Error bars indicate one standard deviation.

References

1. Wolfe JM (2003) Moving towards solutions to some enduring controversies in visual search. Trends in Cognitive Sciences 7: 70–76.

2. Wolfe JM, Friedman-Hill SR, Stewart MI, O'Connell KM (1992) The role of categorization in visual search for orientation. Journal of Experimental Psychology: Human Perception and Performance 18: 34–49.
