# Supplementary material for: Visual Feature Integration Indicated by pHase-Locked Frontal-Parietal EEG Signals
Source: PLoS One. 2012 Mar 9;7(3):e32502. doi: 10.1371/journal.pone.0032502 (PMC3302878; doi:10.1371/journal.pone.0032502)
Supplement: Text S3 — Visual search and fibred products. (PDF) [file pone.0032502.s003.pdf]

# Text S3

A *category theory* [1] treatment of information integration for inference tasks was provided in [2]. The basic idea is that differences in children's ability to make certain inferences depends on their capacity to compute *(co)products* of a given arity (i.e., number of arguments), where arity is interpreted as the number of sources of information to be integrated. So, for example, a task (or, task condition) that requires a binary product is formally different (in the category-theoretic sense) from a task (or condition) that requires only a unary product. The purpose here is to show how this category-theoretic approach also applies to visual search, and thereby provides a link both to changes in brain synchrony and children's cognitive development.

The treatment provided here is brief, since the motivation and other formal details are already available in [2]. Only those aspects specific to visual search are presented here. The application to visual search uses the formal category theory concept of *fibred product* (also called *pullback*), so its definition and that of *category* are given. (See [2] for an application of the closely related concept of *fibred coproduct*, also called *pushout*.) In certain situations, a fibred product of some arity is reducible to one of a lower arity. This situation is analogous to multiplication, where for example $x \times 1 = x$. Again, see [2] for examples relating to inference. Reducibility in the context of visual search is shown here.

## Category

A *category* $\mathbf{C}$ consists of a class of objects $|\mathbf{C}| = (A, B, \ldots)$; a set $\mathbf{C}(A, B)$ of morphisms (also called arrows, or maps) from $A$ to $B$ where each morphism $f : A \to B$ has $A$ as its *domain* and $B$ as its *codomain*, including the *identity* morphism $1_A : A \to A$ for each object $A$; and a composition operation, denoted "$\circ$", of morphisms $f : A \to B$ and $g : B \to C$, written $g \circ f : A \to C$ that satisfies the laws of:

- *identity*, where $f \circ 1_A = f = 1_B \circ f$, for all $f : A \to B$; and
- *associativity*, where $h \circ (g \circ f) = (h \circ g) \circ f$, for all $f : A \to B$, $g : B \to C$ and $h : C \to D$.

For concreteness, the application to visual search (below) refers to the category **Set**, which has sets for objects and set-valued functions for morphisms, where the identity morphisms are identity functions, and the composition operator is just function composition.

## Fibred product (pullback)

A *fibred product (pullback)* of morphisms $f : A \to C$ and $g : B \to C$ in a category **C** is an object $P$ and a pair of morphisms $p_1 : P \to A$ and $p_2 : P \to B$ satisfying $f \circ p_1 = g \circ p_2$, such that for any pair of morphisms $z_1 : Z \to A$ and $z_2 : Z \to B$ such that $f \circ z_1 = g \circ z_2$, there is a unique morphism $u : Z \to P$, such that the following diagram *commutes*:

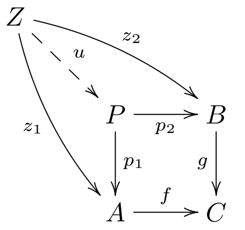


(1)

Such a pullback object $P$ may also be denoted by $A \times_C B$. The constraint is contained in the requirement that the square in Diagram 1 should commute. (A diagram *commutes* when all paths that share the same starting object and the same finishing object are equal. For example, in Diagram 1, the two paths from object $P$ to object $C$ are equal, i.e., corresponding function composition $f \circ p_1 = g \circ p_2$.)

For the application to visual search, a fibred product in **Set** is a Cartesian product of sets $A$ and $B$ constrained at object $C$, sometimes written $A \times_C B$, consisting of all pairwise combinations of $A$ and $B$ such that $f \circ p_1(a, b) = g \circ p_2(a, b)$, i.e., $f(a) = g(b)$, where $(a, b) \in A \times B$.

## Visual search: Display sets and fibred products

The standard model of vision [3] supposes that items are perceived via feature maps, where features of items (such as colour) are represented in some topographical map that preserves location information of the perceived items. Thus, distinguishing a red-vertical bar from a blue-horizontal bar requires integrating two sources of information: the colour feature map and the orientation feature map constrained by common location, so as not to misrepresent the items as a red-horizontal bar or a blue-vertical bar.

This integration process is modeled as a binary fibred product in category theory, as indicated in the

following commutative diagram:

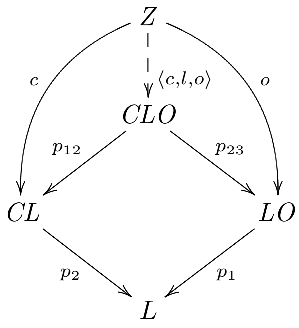


(2)

where $CLO$ is the fibred product object integrating colour and orientation, $CL$ is the colour-location map, $LO$ is the orientation-location map, and $L$ is the constraining location object. (Note that $CLO$ is just $CL \times_L LO$ with the redundant features at $L$ removed.) Morphisms $p_1$, $p_2$, $p_{12}$, and $p_{23}$ are projections, i.e., a projection $p_i$ returns the element at position $i$ of a tuple of elements, and a projection $p_{ij}$ returns the elements at positions $i$ and $j$ (in that order) of a tuple of elements.

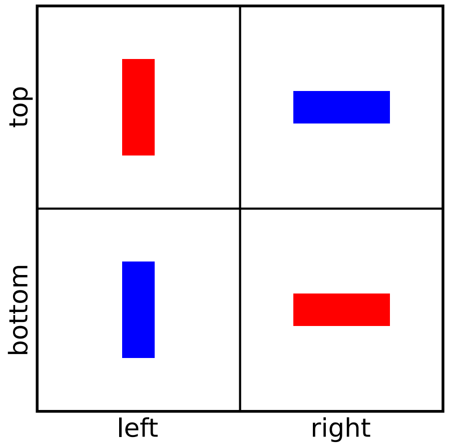


**Figure 1.** Example display set for conjunctive search condition, where arity is binary.

A concrete example using a display set for a conjunctive search (binary arity) condition is shown in Figure 1. For conciseness, reference to the frequency feature is omitted, locations are numbered: top-left (0), top-right (1), bottom-left (2), and bottom-right (3); colours are labelled: red ($r$), blue ($b$), green ($g$), and yellow ($y$); and orientations are labelled: vertical ($v$), and horizontal ($h$). The corresponding fibred

product is indicated in the following commutative diagram:

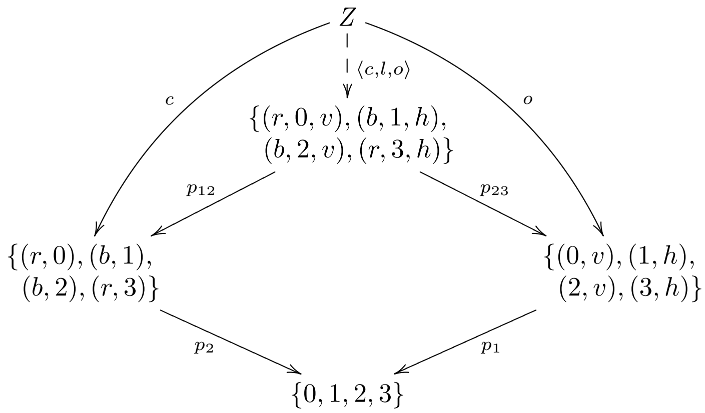


(3)

where, for example, $p_{12} : (r, 0, v) \mapsto (r, 0)$, $p_2 : (r, 0) \mapsto 0$, and $p_{23} : (r, 0, v) \mapsto (0, v)$, $p_1 : (0, v) \mapsto 0$, so $p_2 \circ p_{12} = p_1 \circ p_{23}$. Having integrated colour and orientation maps, the location of a target item (say, red-vertical bar) is identified by composing a morphism (not shown) from an object containing the target to the object containing the integrated information (e.g., $t : (r, v) \mapsto (r, 0, v)$) with a morphism from that object to the location object (i.e., $p_2 \circ p_{12} = p_1 \circ p_{23}$). That is, $(p_2 \circ p_{12}) \circ t : (r, v) \mapsto 0$.

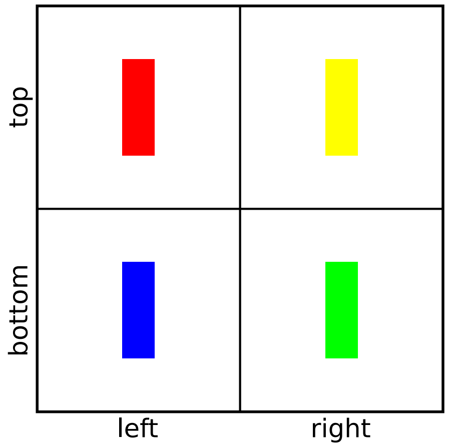


**Figure 2.** Example display set for feature search condition, where arity is unary.

A concrete example using a display set for a feature search (unary arity) condition is shown in Figure 2,

and the corresponding fibred product is indicated in the following commutative diagram:

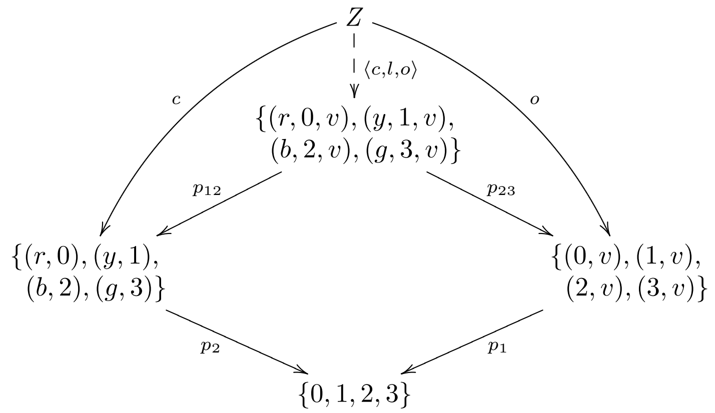


(4)

In Diagram 4, the orientation map object is isomorphic to the location object, and hence object $LO$ can be replaced by object $L$, in which case morphism $p_1$ is replaced by the identity morphism $1_L$. In such situations, the diagram reduces to the following:

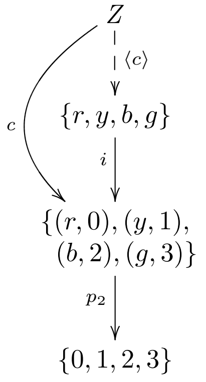


(5)

where $i$ is an injection, e.g., $i : r \mapsto (r,0)$. That is, a unary fibred product involving just the morphism $i$ and the constraint morphism $p_2$. So, computing the location of each object only requires "integrating" one source of information, the colour map in this example. In this case, the target location is retrieved by composing $t'$ (e.g., $t' : r \mapsto r$) with $p_2 \circ i$. That is, $(p_2 \circ i) \circ t' : r \mapsto 0$. So, only one source of information (colour) is needed.

### Other categories

**Set** is not the only category having products. Examples of other categories that have products are **Top**, the category of topological spaces (objects) and continuous functions (morphisms), and **Met**, the category of metric spaces and continuous functions. The objects in these categories also have internal structure, such as a distance measure in the case of **Met**. Such categories may be useful for modeling additional aspects of visual search, such as possible distance effects. In **Met**, for example, a product object is a product metric space.

## References

1. Mac Lane S (2000) Categories for the working mathematician. Graduate Texts in Mathematics. New York, NY: Springer, 2nd edition.

2. Phillips S, Wilson WH, Halford GS (2009) What do Transitive Inference and Class Inclusion have in common? Categorical (co)products and cognitive development. PLoS Computational Biology 5: e1000599.

3. Riesenhuber M, Poggio T (2004) How the visual cortex recognizes objects: The tale of the standard model, Cambridge, MA: MIT Press, volume 2, chapter 111. pp. 1640–1653.
